# Early Dissemination of Circulating Tumor Cells: Biological and Clinical Insights

**DOI:** 10.3389/fonc.2021.672195

**Published:** 2021-05-07

**Authors:** Francesca Chemi, Sumitra Mohan, Tatiana Guevara, Alexandra Clipson, Dominic G. Rothwell, Caroline Dive

**Affiliations:** Cancer Research UK Manchester Institute Cancer Biomarker Centre, University of Manchester, Macclesfield, United Kingdom

**Keywords:** liquid biopsy, CTCs, early dissemination, metastasis, minimal residual disease

## Abstract

Circulating tumor cells (CTCs) play a causal role in the development of metastasis, the major cause of cancer-associated mortality worldwide. In the past decade, the development of powerful cellular and molecular technologies has led to a better understanding of the molecular characteristics and timing of dissemination of CTCs during cancer progression. For instance, genotypic and phenotypic characterization of CTCs, at the single cell level, has shown that CTCs are heterogenous, disseminate early and could represent only a minor subpopulation of the primary tumor responsible for disease relapse. While the impact of molecular profiling of CTCs has not yet been translated to the clinic, CTC enumeration has been widely used as a prognostic biomarker to monitor treatment response and to predict disease relapse. However, previous studies have revealed a major challenge: the low abundance of CTCs in the bloodstream of patients with cancer, especially in early stage disease where the identification and characterization of subsequently “lethal” cells has potentially the greatest clinical relevance. The CTC field is rapidly evolving with development of new technologies to improve the sensitivity of CTC detection, enumeration, isolation, and molecular profiling. Here we examine the technical and analytical validity of CTC technologies, we summarize current data on the biology of CTCs that disseminate early and review CTC-based clinical applications.

## Introduction

The major cause of cancer related mortality is metastasis (1, 2) which is attributed to dissemination of cancer cells, referred to as circulating tumor cells (CTCs), from the primary site *via* the bloodstream or the lymphatic system to subsequently form secondary tumors in distant sites. The burden of CTCs is strongly associated with cancer prognosis in several cancer types (3). Metastasis was long thought to occur in the later stages of cancer progression in patients with advanced disease. However, a growing body of evidence reports the presence of CTCs at earlier stages of tumor growth, even before the detection of primary tumor (4, 5). CTCs may seed active metastatic tumors or remain in a latent state called tumor cell dormancy that, at some point in time and *via* mechanisms incompletely defined, exit dormancy to form metastases (6, 7). Comprehensive analysis of CTCs is central to understanding mechanism(s) of cancer metastasis. Molecular profiling of CTCs, and particularly early disseminating CTCs could also lead to discovery of new prognostic and predictive biomarkers to inform patient management. The major challenge for CTC detection and analysis is their rarity in a typically sampled blood volume (10-50ml), and this is observed in most patients with advanced cancer with some notable exceptions such as Small Cell Lung Cancer (8, 9). This challenge of low CTC prevalence is further magnified in early disease settings. In addition to their rarity, CTCs are also genetically and phenotypically heterogeneous (including the spectrum of epithelial to mesenchymal phenotypes). Technologies that can accommodate CTC heterogeneity are critically needed. This mini review will focus on early disseminating tumor cells; in particular, we will summarize methods for identification and characterization of CTCs and give an overview of current knowledge on the biology and clinical relevance of CTC early dissemination.

## Identification and Characterization of CTCs

CTC enumeration is a well-established biomarker for cancer diagnosis, prognosis, disease progression and prediction of therapeutic response (10). CTCs can be separated and/or enriched from other blood cells by using different technologies that exploit either their physical properties (e.g., size, weight, density, deformability, electrical), or differential expression of molecular markers (commonly intracellular and surface protein expression) or a combination of both (11–15). CTC enrichment techniques employing affinity-based capture methods use antibodies binding to cell surface markers and are based on two strategies; 1) the negative enrichment approach that employs upstream immunomagnetic depletion to remove CD45-positive White Blood Cells (WBCs), though this is rarely achieved to completion, and 2) the positive enrichment approach that captures CTCs and then subsequently removes WBCs (14, 16). While positive enrichment fails to capture cells with low or negative expression of the CTC markers, negative enrichment strategies typically have a lower stringency compared to positive enrichment. The commercial RosetteSep™ CTC Enrichment Cocktail (StemCell Technologies) integrates negative immunoaffinity-based enrichment with density centrifugation. The technology utilizes tetrameric antibody complexes against cell surface antigens found on human hematopoietic cells (CD2, CD16, CD19, CD36, CD38, CD45, and CD66b) and glycophorin A that enables the removal of white and red blood cells from whole blood, thereby enriching for the remaining CTCs (17). Among the positive enrichment approaches, CellSearch^®^ technology is based on immunomagnetic enrichment which uses an epithelial cell adhesion molecule (EpCAM) coated on ferromagnetic particles, with subsequent immunomagnetic separation (18). Alternative CTC isolation techniques are required to capture mesenchymal CTCs or CTCs in the dynamic process of EMT. Additional CTC platforms using enrichment technology include those based on immunomagnetic separation such as a magnetic cell separation system (MACS) (19) and CTC-Chip which still employs an EpCAM-based enrichment approach combined with a microfluidic device (16, 20). The most commonly used antibodies to identify epithelial cells are EpCAM and cytokeratins (CK-19). However, CTCs undergoing epithelial–mesenchymal-transition (EMT) can gradually lose their epithelial characteristics, having no or very low expression of EpCAM and consequentially evade capture and increasingly CTC platforms are combining epithelial markers with others markers, such as mesenchymal markers (e.g., N-cadherin, vimentin or TWIST1), stem cell markers (CD133, CD44, CD34, ALDH1) (21) early apoptosis markers (M30, Bcl-2) or cancer specific markers (e.g., HER2, PSMA) (22, 23). Another platform for CTC detection is AdnaTest (Adnagen AG) that enriches CTCs using a cocktail of antibodies (e.g., EpCAM, MUC-1, AR, Her2) specific to the cancer type (e.g., breast, lung, prostate, ovarian) followed by a subsequent analysis of tumor associated gene expression by RT-qPCR (24). More recently, an approach using an *in vivo* positive enrichment technology named GILUPI CellCollector^®^ (GILUPI Nanomedizin) has been described. This technology allows capture of CTCs directly from the cubital vein of the patient by using antibodies against EpCAM with the advantage of using the total volume of blood and increasing the chance of CTCs isolation (18).

CTCs can be enriched without labelling based on their physical properties. Tumor cells are normally (but not always) larger than most blood cells and this characteristic has been exploited to capture CTCs by size-based filtration using of microfluidic device/cartridges or microchips to separate cells. One option for density-based CTC enrichment is the AccuCyte assay which uses a density-gradient separation technology that integrates a separation tube and a collector device (25). Another example of CTC enrichment technology based on physical properties is the Parsortix™ system that traps cells based on both deformability and size in disposable cassettes with channels that gradually decreases in size to approximately <10μm (26). A combinatory strategy using a microfluidic platforms and a nanotechnology-assisted separation has been also developed for CTC isolation (27). Alternative methods using microfiltration-based enrichment which isolate tumor cells by size include ISET^®^ (Rarecells Diagnostics) (17) and ScreenCell^®^ (28). The electrical properties of CTCs can also be used for tumor-cell isolation by applying a non-uniform electric field through the phenomenon of dielectrophoresis (DEP). Here, a positive (pDEP) or negative (nDEP) force is applied to a cell, moving it towards or away from the electric field source, respectively. Some systems have been described which employ DEP enrichment technologies including ApoStream^®^ (29) and DEParray™ automated system. The latter traps stained cells in DEP cages and is combined with a high-resolution imaging device. Single cells, selected *via* marker expression can be moved within the chip by electrical forces and physically isolated for further genomic analysis (30).

Given the likely loss of CTCs with any enrichment step, CTC platforms have been designed to capture all nucleated cells in the bloodstream. The high-definition single-cell assay (HD-SCA) developed in Peter Kuhn’s laboratory (31, 32) and commercially available through Epic Sciences, is based on such a ‘no cell left behind’ approach, where the entire population of cells in a liquid sample are plated as a biological monolayer onto glass slides and immune-stained for HD-CTC identification. Each slide subsequently undergoes sophisticated image processing to detect rare cells that can be physically picked for genomic analysis or subjected to single cell CYTOF to assess up to 40 proteins per CTC (12, 31, 33). For single CTC image analysis, different tools have been developed for automated processes of classification, sorting, and detection of CTCs for subsequent genetic analysis. A recent study introduced an analysis program called ACCEPT, which classified fluorescent images of single cells from CellSearch^®^ platform as CTCs or not CTCs with an accuracy of 96% (34). In contrast, an automatic tool for label-free CTC detection is also possible whereby CTCs and WBCs are identified directly from bright field microscopy images (35). We cannot describe all the currently available CTC platforms, but refer the reader to a comprehensive recent review (36).

Not all cells detected that are classified phenotypically as CTCs carry genomic aberrations (30, 37, 38) and increasingly phenotypic identification of CTCs is followed by molecular profiling to confirm whether circulating cells, however enriched and isolated, are tumor cells. Molecular profiling of CTCs could also provide unprecedented windows onto the metastatic process, underlying tumor heterogeneity and information on treatment response and resistance (17, 29, 39–44). With the evolving field of single-cell technologies, evaluation of DNA, RNA and protein alterations at the single cell level is now feasible and is being applied to CTCs (**Table 1**) and analysis of paired primary tumor and CTCs has the potential to shed light onto tumor evolution. A study performed on 23 patients showed that shedding of CTCs from the primary tumor is not random; instead, acquisition of copy number aberrations (CNA) is driven by a convergent process across tumor types that ultimately leads to the release of CTCs with complex genomic rearrangements (56). In another study in breast cancer, CTCs resembled CNA of primary tumors and contained alterations associated with brain metastasis with high clonality, suggesting that brain metastasis competent cells had undergone clonal selection (57). Single cell analysis, although exciting can be limited by failures in the technically challenging steps within the workflows. For this reason, expanding CTCs in 2D or 3D cultures or *via in vivo* models could overcome the technical limitations of single CTC analysis and facilitate functional studies. Primary cultures from CTCs have been successfully established in patients with advanced stage cancer (58) which maintained molecular and phenotypic properties of the uncultured primary CTCs, matched genetic alterations of the corresponding primary tumor and could be used to assess molecular changes over time with serial blood draws (59). In contrast, low success of CTC cultures has been reported for patients with early stage cancers, most likely due to the lower abundance of CTCs compared to patients with advanced stage cancer. Optimization of culture conditions and development of eventually CTC cell lines is thus a worthy goal that will improve our understanding of the biological properties of early disseminating tumor cells.

**Table 1:** Summary of studies that performed CTC molecular profiling.

Molecular type	Technology	Readout	Type of Cancer	Main conclusions	References
**Genome**	Array-CGH/targeted NGS	CNA/mutations	Colorectal	1) CTCs carry tumour CNA and mutations 2) CTCs represent a small subclone of primary tumour	[Heitzer et al. (45)]
	WES/WGS	CNA/mutations	Lung	1) CTCs carry heterogeneous mutation patterns 2) CNAs are reproducible within CTCS and are selected to lead metastasis	[Ni et al. (46)]
	WES	Mutations	Prostate	1) Feasibility of sequencing whole exome from single CTCs 2) CTCs carry early mutations in tumour evolution	[Lohr et al. (47)]
	WGS	CNA	Lung	1) CNA profiles from single CTCs predict patient’s chemosensitivity 2) The CNA classifier correctly assigns 83.3% of the cases as chemorefractory or chemosensitive	[Carter et al. (39)]
	Targeted NGS	Mutations	Breast	1) Mutational heterogeneity in PIK3CA, TP53, ESR1, and KRAS genes between individual CTCs 2) cfDNA profiles provided an accurate reflection of mutations seen in individual CTCs	[Shaw et al. (48)]
	WES	Mutations	Lung	1) CTCs isolated at early stage cancer carry mutation profiles more similar to the metastasis detected 10 months later 2) Potential of using CTCs to predict metastatic genetic lansdscape in early stage lung cancer	[Chemi et al. (30)]
	Targeted NGS	Mutations	Lung	1) CTCs from ALK-rearranged patients resistant to crizotinib are heterogenous 2) Sequencing CTCs at the single-cell level enables to identify resistance mutations	[Pailler et al. (17)]
**Transcriptome**	RNA *in situ* hybridization	Gene expression	Breast	1) Mesenchymal cells are highly enriched in CTCs 2) Serial CTC monitoring suggests an association of mesenchymal CTCs with disease progression	[Yu et al. (40)]
	RNA-Seq	Gene expression	Prostate	1) CTCs from prostate cancer patients show heterogeneous gene expression patterns 2) Activation of noncanonical Wnt signaling in CTCs from patients progressing under treatment	[Miyamoto et al. (41)]
	RNA-Seq	Gene expression	Breast	1) 17-gene digital signature of CTC–derived transcripts enable high-sensitivity early monitoring of response 2) CTC-RNA signatures may help guide therapeutic choices in localized and advanced breast cancer	[Kwan et al. (49)]
	Padlock probe technology	Gene expression	Prostate/Pancreas	1) Quantification of AR-V7, AR-FL, PSA, and KRAS mut/wt transcripts in CTCs 2) Padlock probe technology compatible with multiple CTC-isolation devices	[El-Heliebi et al. (50)]
	Whole-genome microarray	Gene expression	Melanoma	1) Melanoma CTCs at advanced disease stages contain heterogeneous cell pools bearing distinct characteristics associated with bone marrow 2) Transcriptional subtyping of melanoma CTCs provides key insights into the molecular mechanisms that regulate metastatic potency	[Vishnoi et al. (51)]
	RNA-Seq	Gene expression	Breast	1) Neutrophils directly interact with CTCs to support cell cycle progression in circulation and to accelerate metastasis seeding 2) CTC–neutrophil clusters may be targeted therapeutically	[Szczerba et al. (44)]
**Epigenome**	Methylation-specific PCR	Gene specific methylation	Breast	1) Breast cancer metastasis suppressor-1 (RSM1) promoter methylation was detected in a subset of CTCs 2) RSM1 promoter methylation status has biomarker potential in breast cancer	[Chimonidou et al. (52)]
	Multiplex PCR on bisulfite treated DNA	Gene methylation	Breast/Prostate	1) Hypermethylation at promoters of key EMT genes is not frequent in CTCs 2) Epigenetic heterogeneity among CTCs	[Pixberg et al. (53)]
	Whole genome bisulfite sequencing	Global methylation	Breast	1) Hypomethylation of binding sites for stemness and proliferation associated transcription factors in CTC clusters 2) Na+/K+ ATPase inhibitors enable the dissociation of CTC clusters into single cells	[Gkountela et al. (43)]
	ATAC-Seq	Chromatin accessibility	Breast	1) CTC lines established from breast cancer patients generate metastases in mice with similar pattern as seen in corresponding patients 2) MYC is a crucial regulator for the adaptation of DTCs to the activated brain microenvironment	[Klotz et al. (42)]
**Proteome**	Antibody barcode microarray	Intracellular proteins	Lung	1)Eight intracellular proteins were measured in more than 80% of CTCs 2) This method is suitable for co-detection of glucose up-take, intracellular proteins, and mutations	[Zhang et al. (54)]
	Single cell western blot	Multiple protein targets	Breast	1)A protein panel comprising of specific targets for breast cancer can distinguish CTCs from WBCs 2) Targeted proteomic methodology is a promising approach for identifying new CTC targets of interest	[Sinkala et al. (55)]
	Single cell mass cytometry	Multiple protein targets	Prostate	1) Approach that enables multiplex proteomic profiling in addition to morphometric and genomic characterization of CTCs 2) Samples stored for several years can be revisited and analyzed de novo as new protein targets are identified	[Gerdtsson et al. (33)]

This table summarises some of the key studies in the field molecular profiling of CTCs that have shaped our understanding of the potential of CTCs in the clinic as well as for dissecting the biology of cancer. WGS, whole genome sequencing; WES, Whole exome sequencing; CNA, Copy number analysis; NGS, Next-generation sequencing; RNA-Seq, RNA sequencing; PCR, polymerase chain reaction.

## CTCs as Precursors of Metastasis

**Figure 1:**
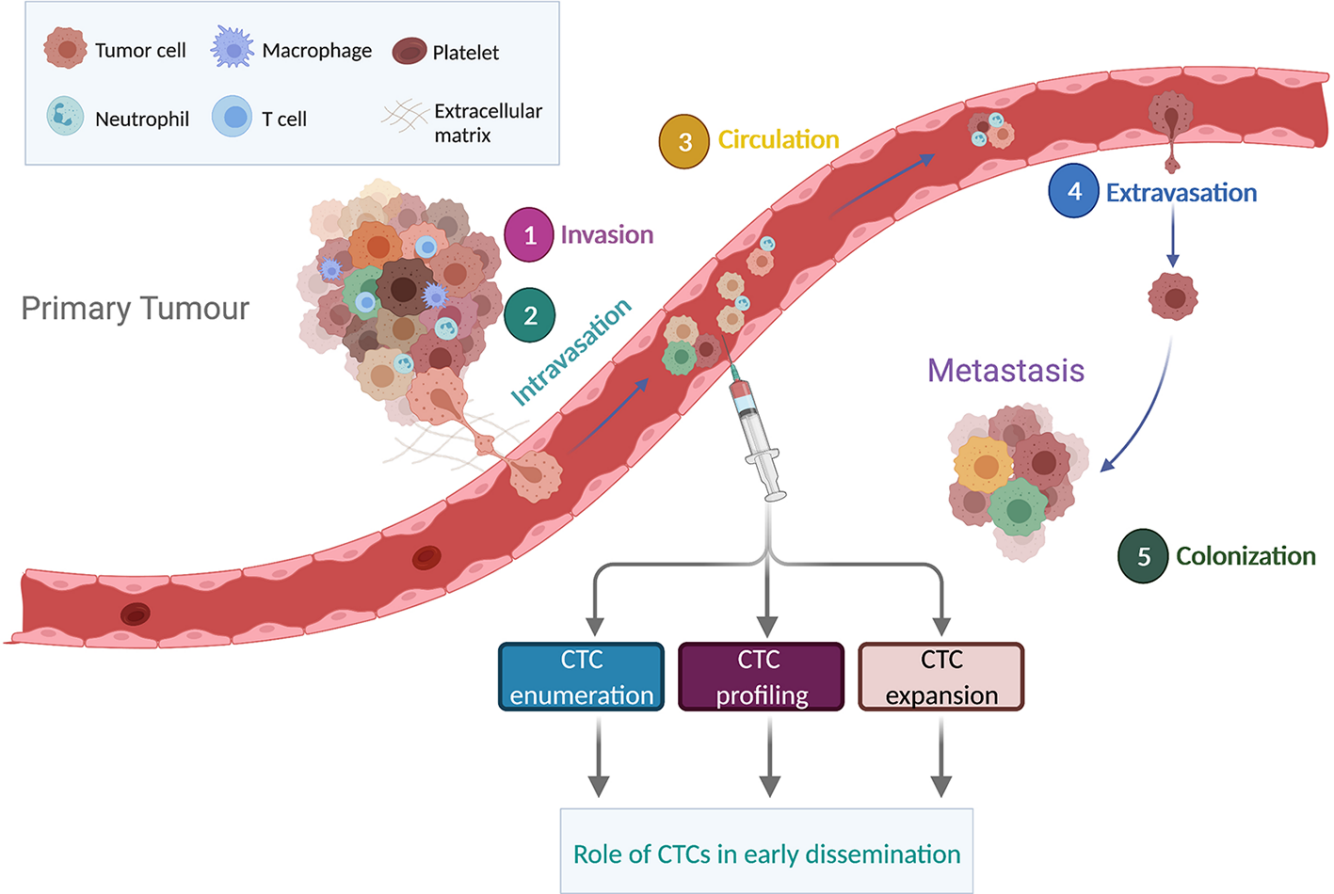
Overview of the metastatic cascade. The metastatic process includes invasion, intravasation, circulation, extravasation and colonization. CTCs detaching from the primary tumor can travel alone or as clusters. Enumeration, molecular profiling and expansion of CTCs in non-metastatic tumors could provide a better understanding on the significance of CTC early dissemination. Figure created in BioRender.com.

Metastasis is a complex, multi-step process *via* which cancer cells leave the primary tumor, intravasate and survive in the bloodstream, extravasate, invade and colonize a secondary organ site before growing into a macroscopic metastatic lesion (**Figure 1**) (60). For epithelial tumors, an early step of the metastatic cascade is proposed to occur *via* a dedifferentiation program known as epithelial-to-mesenchymal transition (EMT). During EMT, tumor cells downregulate epithelial markers such as E-cadherin, detach from neighboring cells and acquire a more invasive mesenchymal phenotype (61). EMT program can be stimulated by multiple factors including an activated tumor associated stroma or under hypoxic conditions (62). In addition, the invasive tumor cells upregulate metalloproteinase activity leading to degradation of extracellular matrix and enabling tumor cell migration to reach the vasculature (63). However, recent studies in mouse models have shown that invasion and metastasis can occur independently of EMT (64–66). In particular, E-cadherin may enhance survival during tumor cell detachment, dissemination and metastatic seeding by limiting reactive oxygen-mediated apoptosis (66). These findings may at least in part explain the prognostic role of epithelial CTCs detected by CellSearch^®^ technology in several cancer types (14) and the presence of hybrid phenotypes (epithelial/mesenchymal) in patients with cancer (67, 68).

Aggressive tumor cells can also transition towards a vascular phenotype by expressing endothelial markers and forming blood vessels, a phenomenon called vasculogenic mimicry (VM) (69). Although VM has been described in breast, ovary, lung, prostate, and bladder cancer and has been associated with dissemination and metastasis, it remains a controversial issue, with concerns including a lack of robust discrimination between VM and endothelial blood vessels (70). However, a subpopulation of small cell lung cancer CTCs that co-expressed VE-cadherin (a marker of VM) and epithelial markers had a copy number profile confirming tumor origin, implying that in this aggressive lung cancer, VM may be causally involved in CTC dissemination (71).

CTCs can travel as single cells or as cell aggregates called CTC clusters or circulating tumor microemboli which have been reported for several cancer types including breast, prostate, lung, and colon cancers (72). Although they are detected at a lower frequency and have significantly shorter half-life in the blood than single CTCs (73), CTC clusters are more likely to form metastasis in mouse models (73). CTC clusters can include non-tumor cell types including pericytes, immune cells, platelets and cancer-associated fibroblasts (74) which may support the survival of the clustered CTCs. A recent study identified neutrophils accompanying CTCs in patients with advanced-stage breast cancer where interactions between neutrophils and CTCs mediated by VCAM1, promoted cell cycle progression and metastatic seeding, opening up new therapeutic vulnerabilities to prevent breast cancer spread (44). Platelets can also interact with CTCs, providing a surrounding ‘shield’ that prevents recognition by the immune system and protects against shear stress forces within the bloodstream (60). RNA-seq performed on single CTCs revealed that platelet markers were highly expressed in a subset of pancreatic CTCs, supporting the interaction of these two cell types in the circulation (75). CTC survival within the bloodstream may also be achieved by up-regulation of β-globin (a subunit of hemoglobin gene normally expressed by red blood cells), as observed in breast, prostate, and lung cancers, with a consequent reduction of oxidative stress within CTCs (76).

Only a minor fraction of CTCs are thought to complete all the steps of the metastatic cascade (77, 78). CTC extravasation is suggested to occur in a similar manner as leucocyte extravasation, a process involving numerous ligands and receptors expressed by both tumor cells and endothelial cells including selectins, integrins, cadherins, CD44 and immunoglobulin (Ig) superfamily receptors (79). The first steps of distant organ colonization may be partially driven by genetic and epigenetic programs present in a subpopulation of tumor cells at any preceding step of the metastatic cascade before CTCs seed metastasis and molecular profiling of sampled CTCs has the potential to uncover their subsequent competencies and perhaps unveil their tissue tropism. Pertinent to this hypothesis, an *in vivo* genome-wide CRISPR screening performed in breast cancer-derived CTCs identified an upregulation of ribosomal proteins and regulators of the translation machinery in a subset of CTCs that associated with high metastatic burden in mouse models (80). Supporting the notion of predicting tissue tropism, comparison of transcriptomic profiles between breast cancer CTCs associated with brain metastasis and CTCs associated with metastasis to other organs revealed a distinct gene signature associated with brain-homing CTCs (81). In addition, CTCs with alterations in metabolic pathways showed a stronger liver tropism in colorectal cancer (82) and protein ubiquitylation was identified as an important mechanism of bone marrow metastatic seeding in melanoma (51).

## CTCs, DTCs and Tumor Dormancy

Once CTCs have survived within the blood stream and extravasated into a distant site they can reside in a dormant state [often referred to as disseminated tumor cells (DTCs)] for years before ‘awakening’ to proliferate and cause overt metastasis (83). Several studies have shown that DTCs can be found in the bone marrow of patients without overt metastases, indicating that these cells disseminate early during tumor progression (84). In support of this hypothesis, genetic analysis of bone marrow DTCs from breast, prostate, and oesophageal cancer revealed fewer chromosomal abnormalities in DTCs than in matched primary tumor cells, indicative of a parallel progression model of metastatic growth (85–87). Identification of DTCs, together with an increased understanding and then targeting of the ‘awakening’ stimuli and mechanism(s) holds potential in improving patients’ clinical outcomes although finding, isolating and analyzing DTCs is technically challenging and invasive. Nevertheless knowledge of tumor dormancy and DTCs has improved in recent years. Extrinsic factors including a lack of angiogenesis, immune surveillance, and the balance between proliferation and apoptosis have all been shown to drive tumor dormancy (88). Molecular profiling of DTCs has enabled a better understanding of the cell-intrinsic signals that induce dormancy, such as inhibition of pathways involved in cell-cycle regulation, metabolic signals and autophagy (89). However, only a few studies have investigated whether CTCs from peripheral blood express markers of tumor dormancy (90–92).

In a breast cancer study, CTC subsets were selected for EpCAM negativity, positivity for stem cell markers (CD44^+^/CD24^−^) and combinatorial expression of uPAR/intβ1 because downregulation of these two markers has been directly implicated in breast cancer dormancy (90). The uPAR^+^/intβ1^+^ subgroup of CTCs were found to be more proliferative compared to uPAR^-^/intβ1^-^ CTCs in *in vitro* assays, suggesting that these two markers could be used to distinguish CTCs that subsequently proliferate *vs* become dormant at distant sites (90). A later study from the same group identified mTOR signaling as a critical determinant in promoting CTC seeding and maintained long-term bone marrow-resident breast cancer cell dormancy (91).

The balance between proliferation and apoptosis has been shown to be associated with tumor dormancy (88, 93). In line with this finding, proliferation and apoptosis markers (Ki67, M30) were measured on CTCs derived from patients with breast cancer who were disease-free for at least 5 years or who relapsed more than 5 years after surgery. The study found that apoptotic CTCs were detected more frequently in patients who remained disease-free compared to those who experienced late relapse, suggesting that the expression of these two markers could be potentially used to predict escape from dormancy (92). In another study, 36% of patients had detectable CTCs 8 to 22 years after mastectomy without evidence of progressive tumor growth. The authors of this study suggest that this could be associated with a failure to complete the final stages of metastasis, which could be potentially being kept in check by a prevailing apoptosis/proliferation balance that maintains a dormant state in distant sites (94). However, the clinical application of CTC detection in the tumor dormancy context still remains unclear. Future research in this field should focus on the identification of CTC molecular features that could distinguish between cancers that are behaving more aggressively from those that will enter a dormant state.

## Clinical Implication of Early Dissemination

Analysis of CTCs have enhanced our understanding of cancer biology (61) as well as the potential vulnerabilities of the metastatic cascade. The application of CTC based assays in a clinical setting, especially in early stage disease has been challenging, primarily due to the low frequency of CTCs. Questions often debated in the field of CTC research are whether the low number of single cells analyzed (typically less than 10) are sufficient to capture tumor heterogeneity and if this heterogeneity is better captured in a tumor biopsy (95). However, most tumor biopsy procedures sample a single region or limited number of regions of a tumor and it is often difficult to assess whether the aggressive tumor clones have been captured (96). In comparison, CTCs are cells that have undergone the selection process and have already entered the metastatic cascade, though as mentioned previously, only a minor fraction complete it. Whether these CTCs are indeed a better representation of the aggressive tumor clones than tumor biopsy is yet to be determined, especially in early stage cancers. To this end, in the past decade several studies have explored the potential of CTCs in clinical research and the implications of early dissemination of CTCs in patient diagnosis as well as prognosis. Although technologies for profiling these rare single cells have evolved in the recent years, single cell manipulation and analysis (capture, enumeration, molecular profiling, and bioinformatic workflows) will likely need to be simplified, automated and less expensive to become routinely feasible and taken up in the clinic.

The low prevalence of CTCs in early disease (97–100) clearly hinders extensive studies. In patients with early breast cancer, the TREAT-CTC trial was the first to demonstrate the clinical utility of CTCs using the CellSearch^®^ platform. This trial addressed the requirement of additional treatment to eliminate CTCs post adjuvant chemotherapy and CTC screening was performed at the end of adjuvant chemotherapy in 1317 patients with HER2 negative breast cancer. Of the 95 CTC positive patients, 63 were randomly assigned to observation or trastuzumab administration. The trial demonstrated the feasibility of CTC based screening in an adjuvant setting as well as the higher rate of relapse amongst CTC positive patients (101). More recently, in a cohort of 75 patients’ with limited stage SCLC (LS-SCLC, defined as tumor confined within only one lung and/or in the lymph nodes in the mediastinum) the CONVERT trial determined that ≥15 CTCs was as an independent prognostic marker with 60% of patients had detectable CTCs at pre-treatment sampling (102). In localized prostate cancer there was a definite trend towards a positive correlation of CTCs with pathological stage as well as a trend towards prognostic and predictive impact of detecting CTCs with several studies reporting correlations with patient survival and/or disease recurrence post treatment (103–107). A study in 2014 of patients with chronic obstructive pulmonary disease (COPD), found detectable CTCs in some patients, with these patients developing lung nodules 1–4 years later and with four patients diagnosed with invasive adenocarcinoma and a fifth diagnosed with squamous cell carcinoma, demonstrating the predictive value of CTCs in early NSCLC (4). However, the study also reported false positives in three patients who did not develop overt cancer suggesting the need for further validation using broader CTC detection systems in large nationwide screening programs. Furthermore, in stage I-III NSCLC CTCs collected at surgery prior to tumor resection from the draining pulmonary vein were higher in count compared to sampling of the peripheral blood (1–3,093 *vs.* 0–4 CTCs in the peripheral blood) and although a larger study will be required to validate this finding, CTC count was associated with risk of relapse (30, 108, 109). Strikingly, in a case study within this cohort, genomic comparison of individual pulmonary vein CTCs to the resected primary tumor and a secondary tumor which developed 10 months later, revealed that CTCs had more genomic variants in common with the metastasis than the primary tumor implicating early disseminating CTCs as responsible for disease relapse (29). Further studies using this approach to confirm these findings are warranted.

A further potential utility of CTCs, given the data emerging on early dissemination, is as biomarkers of minimal residual disease (MRD) following treatment with curative intent where tumor phenotype and genotype can be assessed as indicators of (aggressive versus indolent) relapse time-course as a complementary approach to ctDNA monitoring (110). The incomplete primary tumor eradication with consequent persistence of residual cells in the form of CTCs or DTCs remains a major challenge in the clinical management of patients with cancer. The detection of MRD after primary curative treatment has the potential to identify high-risk patients who can benefit from additional treatments and monitoring. The role of CTCs in MRD monitoring has been investigated in several cancer types including breast, colorectal, lung, and prostate cancers (111). In these studies, detection of CTCs at a follow-up time point (ranging from 3 months to 5 years post chemotherapy, accordingly to the tumor types) was significantly associated with unfavorable outcomes. In particular, studies from our group showed that the presence of CTCs (measured by CellSearch^®^) after one cycle of chemotherapy was associated with worse overall survival in both patients with NSCLC and SCLC (9, 112). Given the proven clinical relevance of CTCs in the MRD setting, ultrasensitive assays are now required in order to detect small number of cells and to capture a broad range of CTC phenotypes (epithelial, mesenchymal or both). More recently, a distinguishing role between CTCs and DTCs has been reported: patients with detectable CTCs in the MRD setting relapsed earlier compared to those with detectable DTCs only, who showed a later relapse (113, 114).

## Conclusions

In the clinic, analysis of CTCs has been used for prognostic stratification of many solid cancers such as breast, small cell lung, non-small cell lung, colorectal, and prostate cancers as well as to monitor disease progression. However, CTCs as a liquid biopsy have not yet fulfilled their undisputable potential to inform of personalized management of patients with cancer which may even extend to the high bar of earlier detection of cancers. The low number of CTCs in the circulation and the sensitivity of the CTC assays currently in use remain a challenge. To this end, intense efforts have been made around the world to standardize CTC based assays to overcome the technical challenges of enrichment, detection, enumeration, isolation, and NGS analyses and to increase assay sensitivity. Developments in the field of CTC enrichment instruments and NGS analyses have elevated CTC studies, bringing exciting insights into biology, heterogeneity and evolution of tumors and begin to illuminate the pathways that underlie tumor dissemination and subsequent steps of the metastatic cascade. These studies include data on CTC heterogeneity, interactions in the blood stream with other cell types, immune evasion, metastatic potential and organ tropism.

However, several unanswered biological questions remain, such as what causes the dissemination of cells into the circulation, what determines the tropism of these CTCs at a metastatic site and further how and which pathways need to be targeted to curb the metastatic potential of these single cells. The answers to these questions could be very different depending on the primary tumor in question and more research must be done to answer them. Furthermore, more studies in large patient cohorts will need to be designed to address the clinical utility of CTCs beyond single CTC and CTC cluster enumeration so that CTC data can be used for individualized tests for drug susceptibility and investigate predictive biomarkers of response to treatments as well as for earlier detection of disease progression. Although, we have come a long way in CTC research the question remains if CTCs are ready for prime time in the clinic. In our opinion, the standardization of CTC assays along with the combining outputs from other liquid biopsy readouts such as cell free DNA, cell free RNA and circulating proteins will help realize their true potential.

## Author Contributions

FC, SM, and TG drafted the manuscript and AC, DR, and CD evolved the manuscript to the final draft. All authors contributed to the article and approved the submitted version.

## Funding 

This work was supported through Core funding to Cancer Research UK (CRUK) Manchester Institute (A27412), the CRUK Manchester Major Centre Award (A25254), the CRUK Lung Cancer Centre of Excellence (A20465) and a R01 grant from US National Cancer Institute (R01 CA197936). Support was received from the Manchester NIHR Biomedical Research Centre, and the Manchester Experimental Cancer Medicine Centre.

## Conflict of Interest

The authors declare that the research was conducted in the absence of any commercial or financial relationships that could be construed as a potential conflict of interest.
